# Assessment of voltage harmonics’ impact on the maximum load capacity of the power supply transformer for the LHCD system

**DOI:** 10.1038/s41598-024-56366-x

**Published:** 2024-03-15

**Authors:** Hui Chen, Junjia Wang, Hejun Hu, Yiyun Huang

**Affiliations:** 1 Institutes of Plasma Physics, Hefei Institutes of Physical Science, Chinese Academy of Sciences, Hefei, 230031 China; 2 University of Science and Technology of China, Hefei, 230026 China

**Keywords:** THDV, Transformers, LHCD, Winding losses, Maximum loading capability, Electrical and electronic engineering, Energy infrastructure

## Abstract

To study the influence of the voltage total harmonic distortion (THDV) and its spectrum on the harmonic loss factor (*F*_*HL*_) and the maximum allowable load capacity ratio (*I*_*max*_ (*pu*)) for the transformer of the LHCD system, we use MATLAB software to model the LHCD system and study the winding loss and maximum allowed load capacity of the LHCD system transformer when the supply voltage source is sinusoidal and non-sinusoidal, respectively. The calculation is carried out by the method of IEEE standard C57.110 and the method of considering the skin effect. The calculation results of both methods clearly show that the THDV value of the supply voltage has a significant effect on the harmonic dissipation factor (*F*_*HL*_) and the maximum allowable load capacity ratio (*I*_*max*_ (*pu*)) of the transformer. And the calculation method considering the skin effect increases the maximum allowable load capacity ratio of the LHCD system transformer by about 2%. The research results have important reference value for the future retrofit design of LHCD system transformers.

## Introduction

The Experimental advanced superconducting tokamak (EAST) was developed by China based on the latest research achievements of Tokamak at the end of the twentieth century. It is the world's first non-circular cross section and ultra-conducting tokamak^1^. The auxiliary heating system of EAST is mainly composed of four parts: LHCD, ECRH, ICRH, and NBIH, of which the LHCD is the most important method for maintaining heat and controlling thermal plasma^2–4^.

To obtain a stable and high-performance plasma current, EAST built a new LHCD system with a frequency of 4.6 GHz and a power of 6 MW in 2014^5^. The high-voltage power supply for the LHCD system is developed based on Pulse Step Modulation (PSM) switch technology. Because it uses a large number of semiconductor devices, the LHCD system contains a large number of harmonics^6,7^. In addition, when the Tokomak device is working, its pulse power is huge, the reactive power changes sharply, the running time is random, and the superconducting load is special. These factors together lead to a large number of non-sinusoidal or harmonic distortion currents and voltages in the entire Tokomak system. Transformers supplying high-voltage power supplies are also exposed to harmonics for long periods of time^8,9^. These harmonics will cause a lot of losses in the transformer, make the transformer overheat, reduce the insulation level, and reduce its service life and load capacity^10^.

Since the additional loss of transformer winding (mainly caused by harmonic current) is much larger than the additional loss of iron core (mainly caused by harmonic voltage)^11^, the overheating problem of transformer is mainly caused by the harmonic current. At present, most of the research on the transformer overheating problem is solved by the method of derating^12,13^. Derating means reducing the nonlinear load provided. Their studies are all based on IEEE Standard C57.110 and UL Standard 1561, using the harmonic loss factor (*F*_*HL*_) and *K* factor to determine the maximum allowable load capacity ratio (the max permissible rms non-sinusoidal load current under rated conditions (*I*_*max*_ (*pu*)) of the transformer^10,14^. However, the basic assumption of IEEE Standard C57.110 is that winding eddy current losses for conductors smaller than 3 mm gradually increase with the square of the harmonic frequency, in line with the maximum winding losses. However, this assumption is wrong for conductors of larger dimensions above the fifth harmonic due to the skin effect^15,16^.

In^17^, the influence of harmonics on winding loss and temperature distribution characteristics was studied, but possible solutions or measures were ignored. In^18^, it shows that the load loss of transformers increases with higher harmonic distortion rates and frequencies, emphasizing the need for measures to mitigate the impact of harmonics on transformer performance. But this article does not discuss the potential impact on transformer lifespan, reliability, or overall system stability.

In addition, some studies have found that the supply voltage harmonics also affect the current harmonic distortion of nonlinear loads^19–22^, so the voltage harmonics may indirectly affect the transformer winding losses and the maximum allowable load capacity of the transformer.

In this paper, considering the skin effect, the *F*_*HL*_ and *I*_*max*_ (*pu*) values of dry-type transformers supplying LHCD systems under sinusoidal and non-sinusoidal voltages are studied, and a revised load loss calculation model for transformer harmonic currents is proposed. The aim is to analyze the indirect effects of voltage harmonics on transformer winding losses and maximum allowable load capacity.

## Description of HVPS system

**Figure 1:**
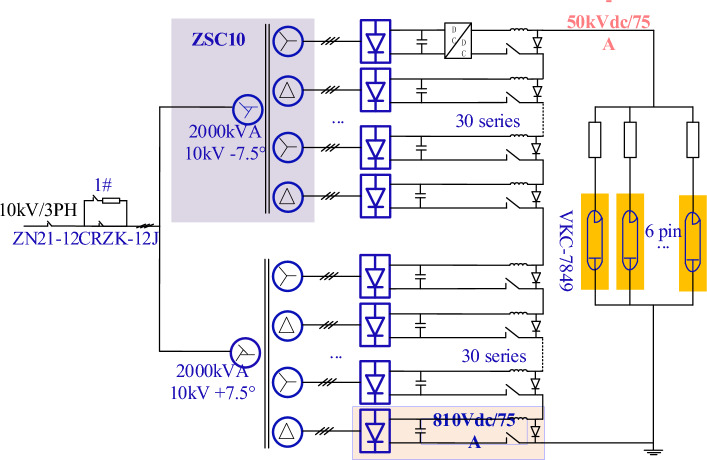
The main circuit configuration of HVPS.

The 4.6 GHz/6 MW LHCD system has a total of four identical HVPS systems. For the convenience of illustration, we only select one HVPS system. Its main circuit configuration is shown in Fig. 1. It can be seen from Fig. 1 that the HVPS system is a complex system with a power supply system voltage of 10 kV and a power distribution cabinet. Other main components are a soft start switch cabinet, multi-winding rectifier transformer, PSM power supply, and control unit. The three-phase 10 kV AC power grid controls the incoming lines of two 2000 kVA rectifier transformers through a 10 kV power distribution cabinet with soft-start switchgear.

Each rectifier transformer provides power for 32 sets of identical PSM modules, and the output voltage of each PSM power module is 810VDC. In order to obtain higher DC output voltage, 64 groups of power modules are connected in series. By controlling the IGBTs of 64 PSM module power supplies, the maximum output voltage of the HVPS system can reach − 50 kV^3,7^. The main parameters of transformers are shown in Table 1.

**Table 1:** The electrical parameters of the transformer.

Item	Parameter
Type	ZSC10-2000/10/32 × 0.6
Rated Power	2000kVA
Rated Current	115/60.1 × 32A
Rated Voltage	10,000/600 × 32V
Rated Frequency	50Hz
No-load Loss	5950W
Load Loss	17740W
I^2^R	15359W
*P*_*EC-R*_	0.155
Eddy current loss	2381

## Derating calculation method

### IEEE standard C57.110 derating method

According to the definition of IEEE standard C57.110 3.1, the value of transformer loss consists of three parts, which are no-load loss, load loss, and total loss. No-load loss is equal to excitation loss, load loss is equal to impedance loss, and total loss is equal to the sum of no-load loss and load loss. The load loss consists of *I*^*2*^*R* loss and stray loss. The Stray loss can be calculated by subtracting resistance loss from measured impedance loss. The stray losses are further divided into winding eddy-current losses (*P*_*EC*_) and stray losses in components other than the windings (*P*_*OSL*_). Then the total load loss can be expressed as Eq. (1):

1 $$P_{LL} = P + P_{EC} + P_{OSL}$$

where *P*_*LL*_ represents the load loss, *P*_*EC*_ represents the winding eddy-current loss, *P*_*OSL*_ represents the other stray loss, *P* is the *I*^*2*^*R* loss portion of the load loss. *P* can be calculated by multiplying the winding resistance *R* by the square of the total rms value of the load individual harmonic currents (*I*_*h*_):

2 $$P = R\sum\limits_{{\text{h}}} {I_{h}^{2} }$$

In IEEE standard C57.110, to convenient perform the calculations in practice, Eq. (1) applied to rated load conditions is rewritten on a per-unit basis as follows in Eq. (3):

3 $$P_{LL - R} \left( {pu} \right) = 1 + P_{EC - R} \left( {pu} \right) + P_{OSL - R} \left( {pu} \right)$$

where *P*_*LL-R*_ (*pu*) is the per-unit load loss under rated conditions, *P*_*EC-R*_ (*pu*) is the per-unit winding eddy-current loss under rated conditions, *P*_*OSL-R*_ (*pu*) is the per-unit other stray loss under rated conditions.

For any defined non-sinusoidal load current, the winding eddy-current loss *P*_*EC*_ can be expressed as:

4 $$P_{EC} = P_{EC - R} \sum\limits_{h = 1}^{h = h\max } {\left( {\frac{{I_{h} }}{{I_{R} }}} \right)}^{2} h^{2}$$

where *h* is the harmonic order, *h*_max_ is the highest significant harmonic number, *I*_*h*_ is the rms current at harmonic h (amperes), *I*_*R*_ is the rms fundamental current under rated frequency and rated load conditions.

The equation for the rms current in per-unit form (base current is rated current) under non-sinusoidal load currents can be expressed as:

5 $$I\left( {pu} \right) = \sqrt {\sum\limits_{h = 1}^{h = h\max } {I_{h}^{2} \left( {pu} \right)} }$$

where *I*(*pu*) is the per-unit rms load current, *I*_*h*_(*pu*) is the per -unit rms current at harmonic *h*.

Then the rms value of the nonsinusoidal load current $I$ can be given by:

6 $$I = \sqrt {\sum\limits_{h = 1}^{h = h\max } {I_{h}^{2} } }$$

To convenient the calculation of the power supply capability of a transformer under balanced non-sinusoidal load conditions, IEEE standard C57.110 defines the harmonic loss indicators *F*_*HL*_ and *F*_*HL-STR*_.

7 $$F_{HL} = {{\sum\limits_{h} {h^{2} \left( {\frac{{I_{h} }}{{I_{1} }}} \right)^{2} } } \mathord{\left/ {\vphantom {{\sum\limits_{h} {h^{2} \left( {\frac{{I_{h} }}{{I_{1} }}} \right)^{2} } } {\sum\limits_{h} {\left( {\frac{{I_{h} }}{{I_{1} }}} \right)} }}} \right. \kern-0pt} {\sum\limits_{h} {\left( {\frac{{I_{h} }}{{I_{1} }}} \right)} }}^{2}$$

8 $$F_{HL\_STR} = {{\sum\limits_{h} {h^{0.8} \left( {\frac{{I_{h} }}{{I_{1} }}} \right)^{2} } } \mathord{\left/ {\vphantom {{\sum\limits_{h} {h^{0.8} \left( {\frac{{I_{h} }}{{I_{1} }}} \right)^{2} } } {\sum\limits_{h} {\left( {\frac{{I_{h} }}{{I_{1} }}} \right)} }}} \right. \kern-0pt} {\sum\limits_{h} {\left( {\frac{{I_{h} }}{{I_{1} }}} \right)} }}^{2}$$

Then the *P*_*LL*_ can be recalculated by combining Eqs. (1), (3), (4), (6), (7), and (8), as shown in Eq. (9):

9 $$P_{LL} \left( {pu} \right) = I^{2} \left( {pu} \right)\left[ {1 + F_{HL} P_{EC - R} \left( {pu} \right) + F_{HL\_STR} P_{OSL - R} \left( {pu} \right)} \right]$$

For dry-type transformers, generally do not consider temperature rise due to other stray losses, since the heat generated is dissipated by cooling air. Then Eq. (9) can be recalculated by Eq. (10):

10 $$P_{LL} \left( {pu} \right) = I^{2} \left( {pu} \right)\left[ {1 + F_{HL} P_{EC - R} \left( {pu} \right)} \right]$$

Therefore, the maximum permissible rms non-sinusoidal load current (*I*_*max*_ (*pu*)) for dry-type transformers under rated conditions or the ratio of the transformer’s maximum permissible current (*I*_*max*_) to the rated current (*I*_*R*_) is given by Eq. (11):

11 $${\text{I}}_{max} (pu) = \frac{{{\text{I}}_{max} }}{{{\text{I}}_{R} }} = \sqrt {\frac{{P_{LL - R} (pu)}}{{1 + F_{HL} P_{EC - R} (pu)}}}$$

### Considering skin effect

The main limitation of Eq. (4) is that it only applies to conductors under 3 mm. However, However, when the conductor is large and the frequency flowing through the conductor is high, the resistance of the transformer winding will increase due to the skin effect and proximity effect^22^, resulting in a relatively high error in the calculation result of Eq. (4). To make up for the limitation of Eq. (4), a function is adopted from^23,24^. In this paper, this function is used to improve the harmonic factor for transformers with larger winding conductors, which is shown in Eq. (12).

12 $$F_{HL}^{*} = {{\sum\limits_{h} {\frac{{F\left( {\lambda_{h} } \right)}}{{F\left( {\lambda_{R} } \right)}}} h^{2} \left( {\frac{{I_{h} }}{{I_{1} }}} \right)^{2} } \mathord{\left/ {\vphantom {{\sum\limits_{h} {\frac{{F\left( {\lambda_{h} } \right)}}{{F\left( {\lambda_{R} } \right)}}} h^{2} \left( {\frac{{I_{h} }}{{I_{1} }}} \right)^{2} } {\sum\limits_{h} {\left( {\frac{{I_{h} }}{{I_{1} }}} \right)^{2} } }}} \right. \kern-0pt} {\sum\limits_{h} {\left( {\frac{{I_{h} }}{{I_{1} }}} \right)^{2} } }}$$

where $F\left( {\lambda_{{\text{h}}} } \right)$ is equal to $\frac{1}{\lambda }\frac{\sinh \lambda - \sin \lambda }{{\cosh \lambda { - }\cos \lambda }}$, $\lambda = \frac{T}{\sigma }$ ($T$ is the field strength, and $\sigma$ is the depth of penetration), $\sigma_{R}$ is the depth of penetration at rated power frequency, $\sigma_{{\text{h}}} = \frac{{\sigma_{R} }}{\sqrt h }$ is the depth of penetration under harmonic condition.

The maximum permissible rms non-sinusoidal load current considering skin effect can then be expressed as follows:

13 $$I_{\max } \left( {{\text{pu}}} \right) = \frac{{I_{\max } }}{{I_{R} }} = \sqrt {\frac{{P_{LL - R} (pu)}}{{1 + F_{HL}^{*} P_{EC - R} \left( {pu} \right)}}}$$

## Simulation

The purpose of this study is to analyze the influence of Tokamak (EAST) voltage harmonics on *F*_*HL*_ and $\frac{{I_{\max } }}{{I_{R} }}$ values of the transformers supplying the HVPS. To this end, we built a system simulation model in MATLAB software. The simulation model includes a programmable voltage source, forty-three sets of the same PSM power modules (C = 3 mF, R = 700 Ω, L = 1.1 mH), two identical dry-type transformers, of which ratings 2000 kVA and 10 kV (wye)/(16 * 0.6 (delta) + 16 * 0.6 (wye)), the secondary winding consists of copper strands of 3.65 mm × 11 mm. Each transformer supplies power to 32 sets of PSM power modules. Note that in each experiment, the HVPS system will choose to invest in different numbers of PSM module groups according to the different objectives of each experiment. Since the parameters of the 64 groups of PSM modules are the same, the low-voltage windings of the transformer supplying power to the PSM modules have only two connection modes: wye and delta. Therefore, for the convenience of analysis, we only analyze two of the PSM modules (one powered by the wye low voltage winding and the other by the delta low voltage winding).

### Considering skin effect

In this paper, the PSM module of the delta connection type is simulated under two voltage scenarios. The first scenario is under sinusoidal voltage conditions. The second scenario is under non-sinusoidal voltage conditions (voltage distortion from 0 to 15%).

*Scenario 1* The voltage source is a sinusoidal voltage, and the simulation results in Table 2. The harmonic loss coefficient of winding eddy current *F*_*HL*_ calculated by IEEE STANDARD C57.110 is 5.0608, and the transformer capacity *I*_*max*_ (*pu*) is about 80.45% of its sinusoidal load current capacity. When considering the skin effect, the F^*^_HL_ is 4.5372, and the I^*^_max_ (*pu*) is about 82.35% of its sinusoidal load current capability.

**Table 2:** The simulation results (*F*_*HL*_, F^*^_HL_, *I*_*max*_ (*pu*) and I^*^_max_ (*pu*) for the delta-connected.

*h*	$\frac{{I_{h} }}{{I_{1} }}$	$\left( {\frac{{I_{h} }}{{I_{1} }}} \right)^{2}$	$h^{2} \left( {\frac{{I_{h} }}{{I_{1} }}} \right)^{2}$	$h^{2} \frac{{F\left[ {\lambda_{h} } \right]}}{{F\left[ {\lambda_{R} } \right]}}$	$h^{2} \frac{{F\left[ {\lambda _{h} } \right]}}{{F\left[ {\lambda _{R} } \right]}}\left( {\frac{{I_{h} }}{{I_{1} }}} \right)^{2}$
1	1.0000	1.0000	1.0000	1.0000	1.0000
5	0.3457	0.1195	2.9874	23.3900	2.7950
7	0.1130	0.0128	0.6251	43.2700	0.5520
11	0.0696	0.0048	0.5857	93.2900	0.4516
13	0.0338	0.0011	0.1926	121.5900	0.1385
17	0.0285	0.0008	0.2347	182.8900	0.1485
19	0.0198	0.0004	0.1410	215.7000	0.0842
∑		1.1394	5.7664		5.1699
*F*_*HL*_	5.0608		$I_{\max } \left( {pu} \right)$	0.8045	
$F_{HL}^{*}$	4.5372		$I_{\max }^{*} \left( {pu} \right)$	0.8235	

**Figure 2:**
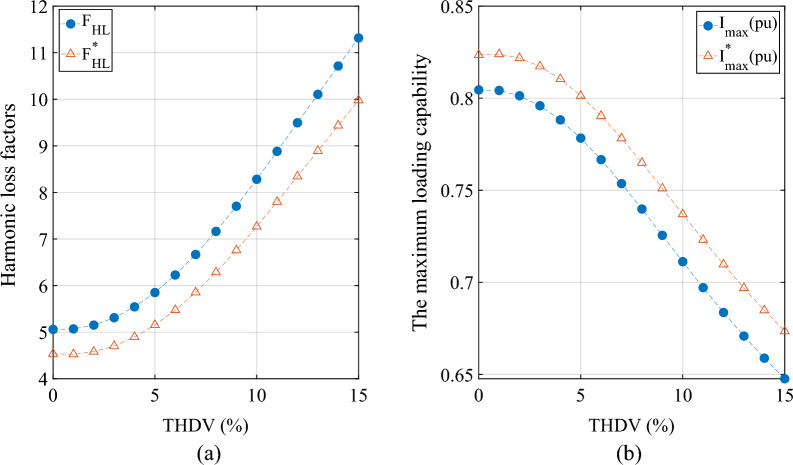
The delta-connected. (**a**) The influence of THDV% on *F*_*HL*_ and  F^*^_HL_. (**b**) The influence of THDV% on *I*_*max*_ (*pu*) and I^*^_max_ (*pu*).

*Scenario 2* In the simulation, under the condition that the power supply is a non-sinusoidal voltage (the seventh harmonic voltage content is 1% to 15% respectively), the values of *F*_*HL*_, F^*^_HL_, *I*_*max*_ (*pu*) and I^*^_max_ (*pu*) are calculated and recorded, and the results are shown in Fig. 2.

### Wye-connected

In this paper, the PSM module of the wye connection type is simulated under two voltage scenarios. The first scenario is under sinusoidal voltage conditions. The second scenario is under non-sinusoidal voltage conditions (voltage distortion from 0 to 15%).

*Scenario 1* The voltage source is a sinusoidal voltage, and the simulation results are tabled in Table 3. From the simulation results, it can be concluded that under the condition of sinusoidal voltage, the harmonic loss coefficient of winding eddy current *F*_*HL*_ calculated by IEEE STANDARD C57.110 is 5.0428, and the transformer capacity *I*_*max*_ (*pu*) is about 80.52% of its sinusoidal load current capacity. When considering the skin effect, the F^*^_HL_ is 4.5192, and the I^*^_max_ (*pu*) is about 82.41% of its sinusoidal load current capability.

**Table 3:** The simulation results (*F*_*HL*_, F^*^_HL_, *I*_*max*_ (*pu*) and I^*^_max_ (*pu*)) of the wye -connected.

*h*	$\frac{{I_{h} }}{{I_{1} }}$	$\left( {\frac{{I_{h} }}{{I_{1} }}} \right)^{2}$	$h^{2} \left( {\frac{{I_{h} }}{{I_{1} }}} \right)^{2}$	$h^{2} \frac{{F\left[ {\lambda_{h} } \right]}}{{F\left[ {\lambda_{R} } \right]}}$	$h^{2} \frac{{F\left[ {\lambda _{h} } \right]}}{{F\left[ {\lambda _{R} } \right]}}\left( {\frac{{I_{h} }}{{I_{1} }}} \right)^{2}$
1	1.0000	1.0000	1.0000	1.0000	1.0000
5	0.3446	0.1188	2.9689	23.3900	2.7777
7	0.1128	0.0127	0.6229	43.2700	0.5501
11	0.0688	0.0047	0.5732	93.2900	0.4420
13	0.0332	0.0011	0.1860	121.5900	0.1338
17	0.0289	0.0008	0.2413	182.8900	0.1527
19	0.0203	0.0004	0.1492	215.7000	0.0891
∑		1.1386	5.7415		5.1454
*F*_*HL*_	5.0428		$I_{\max } \left( {pu} \right)$	0.8052	
$F_{HL}^{*}$	4.5192		$I_{\max }^{*} \left( {pu} \right)$	0.8241	

**Figure 3:**
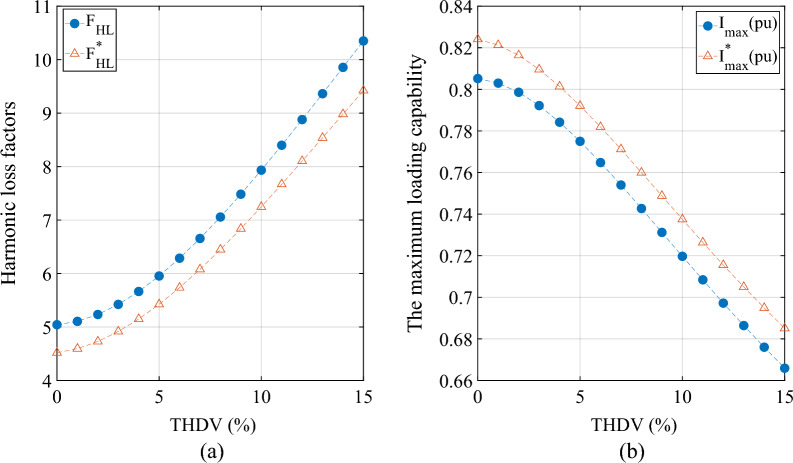
The wye-connected. (**a**) The influence of voltage distortion rate on *F*_*HL*_ and  F^*^_HL_. (**b**) The influence of voltage distortion rate on *I*_*max*_ (*pu*) and I^*^_max_ (*pu*).

*Scenario 2* In the simulation, under the condition that the power supply is a non-sinusoidal voltage (the seventh harmonic voltage content is 1% to 15% respectively), the values of *F*_*HL*_, F^*^_HL_, *I*_*max*_ (*pu*) and I^*^_max_ (*pu*) are calculated and recorded, and the results are shown in Fig. 3.

## Discussion

For scenario 1:

- The results (the wye-connected and the delta-connected) reveal that the percentage error between the eddy current loss factors *F*_*HL*_ and F^*^_HL_ calculated by the two methods is about 9.5% under the condition of sinusoidal voltage.
- The results (the wye-connected and the delta-connected) suggest that the maximum allowable RMS non-sinusoidal load current (*I*_*max*_ (*pu*) and I^*^_max_ (*pu*)) under rated conditions calculated by the two methods differs by about 2%. That is, the calculation method considering the skin effect can increase the maximum allowable rms non-sinusoidal load current under rated conditions by 2%.
- The current practice recommended in the IEEE std.C57 10-2018 standard for evaluating transformers for LHCD systems is conservative.

For scenario 2:

- The results (wye-connected and delta-connected) of the eddy current loss factors *F*_*HL*_ and F^*^_HL_ show that when the voltage distortion rate increases from 1 to 15%, the eddy current loss factors *F*_*HL*_ and F^*^_HL_ calculated by the two methods also increase accordingly and start to increase rapidly from the voltage distortion rate of 3%.

The results (wye-connected and delta-connected) of the maximum allowable RMS non-sinusoidal load current (*I*_*max*_ (*pu*) and I^*^_max_ (*pu*)) show that when the voltage distortion rate increases from 1 to 15%, the maximum allowable RMS non-sinusoidal load current (*I*_*max*_ (*pu*) and I^*^_max_ (*pu*)) calculated by the two methods also decrease accordingly and start to decreases rapidly from the voltage distortion rate of 3%.

## Conclusion

In this paper, the calculation model of transformer loss and maximum allowable current of LHCD system under voltage harmonic environment is established, and the functional relationship between the calculation method of IEEE standard C57.110 and the calculation method considering skin effect is deduced. On this basis, a modified transformer harmonic current load loss calculation model is proposed. Finally, the simulation verification is carried out with Simulink/MATLAB software, and the results show that:

- When the voltage harmonic distortion rate (THDV%) is greater than 3%, the calculation results of both methods show that the harmonic loss factor value of the LHCD system transformer increases significantly and the maximum allowable current value decreases significantly.
- Compare the calculation results of the IEEE Standard C57.110 method with the calculation results of the method considering the skin effect: The harmonic loss factor H value calculated by the former is about 5% higher than that of the latter two. The maximum allowable current F value calculated by the former is nearly 2% lower than that of the latter. The results mean that in the high-frequency environment, the transformer winding loss of the LHCD system is significantly affected by the winding skin effect.

The research in this paper has reference significance for the analysis of transformer losses under the background of voltage harmonics, and can also provide theoretical and data support for the future optimal design and safe operation of LHCD system transformers.

## Data Availability

All data generated or analysed during this study are included in this published article [and its supplementary information files].

## Abbreviations

EAST: Experimental advanced superconducting tokamak
LHCD: Lower hybrid current drive
ECRH: Electron cyclotron resonance heating
ICRH: Ion cyclotron resonance heating
NBIH: Neutral beam injection heating
